# Ginkgolic acid suppresses the development of pancreatic cancer by inhibiting pathways driving lipogenesis

**DOI:** 10.18632/oncotarget.3663

**Published:** 2015-03-26

**Authors:** Jiguang Ma, Wanxing Duan, Suxia Han, Jianjun Lei, Qinhong Xu, Xin Chen, Zhengdong Jiang, Ligang Nan, Jiahui Li, Ke Chen, Liang Han, Zheng Wang, Xuqi Li, Erxi Wu, Xiongwei Huo

**Affiliations:** ^1^ Department of Oncology, First Affiliated Hospital, Xi'an Jiaotong University, Xi'an, China; ^2^ Department of Hepatobiliary Surgery, First Affiliated Hospital, Xi'an Jiaotong University, Xi'an, China; ^3^ Department of General Surgery, First Affiliated Hospital, Xi'an Jiaotong University, Xi'an, China; ^4^ Department of Pharmaceutical Sciences, North Dakota State University, Fargo, ND, USA

**Keywords:** lipogenesis, cancer metabolism, ginkgolic acid (GA), AMP-activated protein kinase (AMPK), pancreatic cancer

## Abstract

Ginkgolic acid (GA) is a botanical drug extracted from the seed coat of *Ginkgo biloba L.* with a wide range of bioactive properties, including anti-tumor effect. However, whether GA has antitumor effect on pancreatic cancer cells and the underlying mechanisms have yet to be investigated. In this study, we show that GA suppressed the viability of cancer cells but has little toxicity on normal cells, e.g, HUVEC cells. Furthermore, treatment of GA resulted in impaired colony formation, migration, and invasion ability and increased apoptosis of cancer cells. In addition, GA inhibited the *de novo* lipogenesis of cancer cells through inducing activation of AMP-activated protein kinase (AMPK) signaling and downregulated the expression of key enzymes (e.g. acetyl-CoA carboxylase [ACC], fatty acid synthase [FASN]) involved in lipogenesis. Moreover, the *in vivo* experiment showed that GA reduced the expression of the key enzymes involved in lipogenesis and restrained the tumor growth. Taken together, our results suggest that GA may serve as a new candidate against tumor growth of pancreatic cancer partially through targeting pathway driving lipogenesis.

## INTRODUCTION

Pancreatic cancer is the fourth leading cause of cancer-related death in the United States with an overall 5-year survival rate less than 6% [1]. In the past, although substantial progress has been made in our understanding of the biology of pancreatic cancer, there is no obvious improvement on survival of this malignancy. Currently, surgical resection offers the only chance to cure pancreatic cancer at early stage. Unfortunately, the vast majority of newfound cases present with advanced unresectable disease at the time of diagnosis, losing the opportunity for radical surgery. Gemcitabine and FOLFIRINOX are the two recommended frontline chemotherapeutic regimens for advanced pancreatic cancer patients [2]. Due to the serious adverse reaction and the disappointed remedial and survival benefits of these chemotherapies, identifying additional novel and effective agents to manage this dreadful disease is of urgent need.

There is growing evidence that metabolic reprogramming plays an important role in cancer development and progression [3]. Elevated fatty acid synthesis is one of the most important alterations of cancer cell metabolism. Previous studies have found that many cancer cells show high rates of *de novo* lipid synthesis, including pancreatic cancer [4], hepatocellular carcinoma [5], breast cancer [6], and prostate cancer [7]. Accumulation of lipid droplets is a frequently observed phenotype in cancer and is a manifestation of abnormal lipid metabolism [8]. A recent study reveals that lipid droplet, as a dynamic organelle, can not only provide energy through β-oxidation for the tumor cells when required, but also play an important role in signaling transduction related with carcinogenesis and cancer cell survival [9]. Inhibition of key enzymes and genes involved in lipogenesis could obviously slow down the growth of tumor cells and impairs their survival [10]. Therefore, to find agents targeting lipogenesis may serve as a promising strategy to treat cancer.

*Ginkgo biloba L*. is an ancient gymnosperm species which is now distributed worldwide, especially in China. Ginkgolic acids (GA), also known as anacardic acids, are mixtures of a series of GA homologues and are the main biologically active ingredients abundantly presented in the leaves and the seed coat of *Ginkgo biloba L*. For many years, a wide range of bioactive properties of GA have been revealed, including anti-HIV [11], anti-bacterium [12], and molluscicidal activities [13]. Recently, the putative anticancer activity of GA is of increasing interest. It has been reported that GA has a potential inhibitory effect on human larynx cancer and tongue squamous carcinoma cells and has no cytotoxic effects on non-tumorigenic cells [14]. However, whether GA has antitumor effect on pancreatic cancer cells and the underlying mechanisms remains unknown. In this study, we investigate the effects of GA on the biological behaviors of pancreatic cancer cells and the underlying potential molecular mechanisms

## RESULTS

### GA inhibits the proliferation of cancer cells

**Figure 1:**
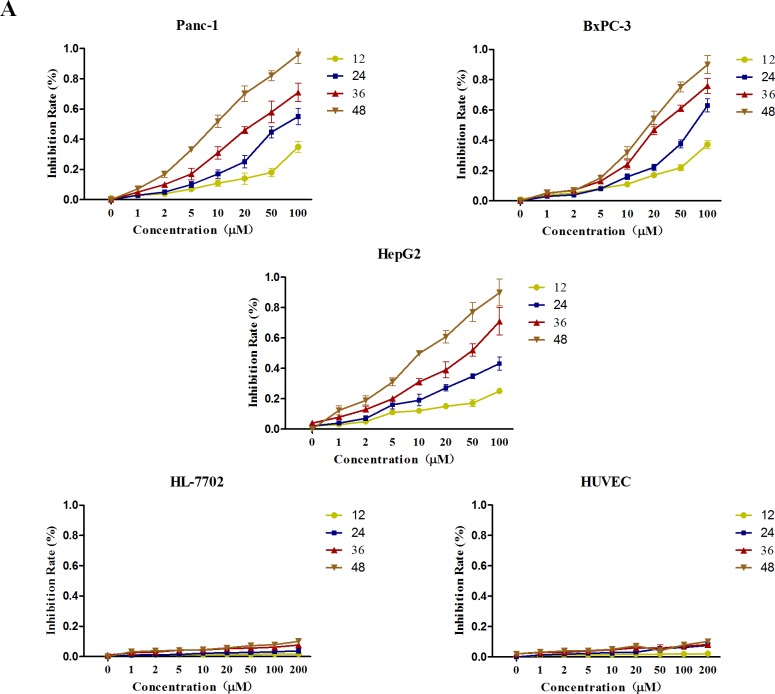
GA treatment suppresses the viability of cancer cells with no cytotoxic effect on non-cancer cells Cancer cells (Panc-1, BxPC-3 and HepG2) and two normal cells (HL-7702 and HUVEC) were treated with various concentrations (0, 1, 2, 5, 10, 20, 50, and 100μM) of GA. At the indicated time points (12, 24, 36, and 48h), cell viability in each group was assessed by MTT assay.

Firstly, we examined the effects of GA on the viability of cancer cell. Pancreatic cancer cell Panc-1, BxPC-3 and hepatocellular carcinoma cell HepG2 were treated with increasing doses of GA (0, 1, 2, 5, 10, 20, 50, and 100μM). Two normal cell lines, HL-7702 (hepatic immortal cell line) and HUVEC (human umbilical vein endothelial cell line) were used as control. At indicated time points (12h, 24h, 36h, and 48h), the cell viability was assessed by MTT assay. As shown in Figure 1, GA decreased the growth of cancer cell lines in a dose- and time- dependent manner; however, limited inhibitory effect on HL-7702 and HUVEC was observed even when we added the GA concentration to 200μM. These results indicated that GA can inhibit the growth of cancer cells with little effect on the normal cells under the concentration of less than 100μM.

### GA inhibits clone formation and induces apoptosis of cancer cells

**Figure 2:**
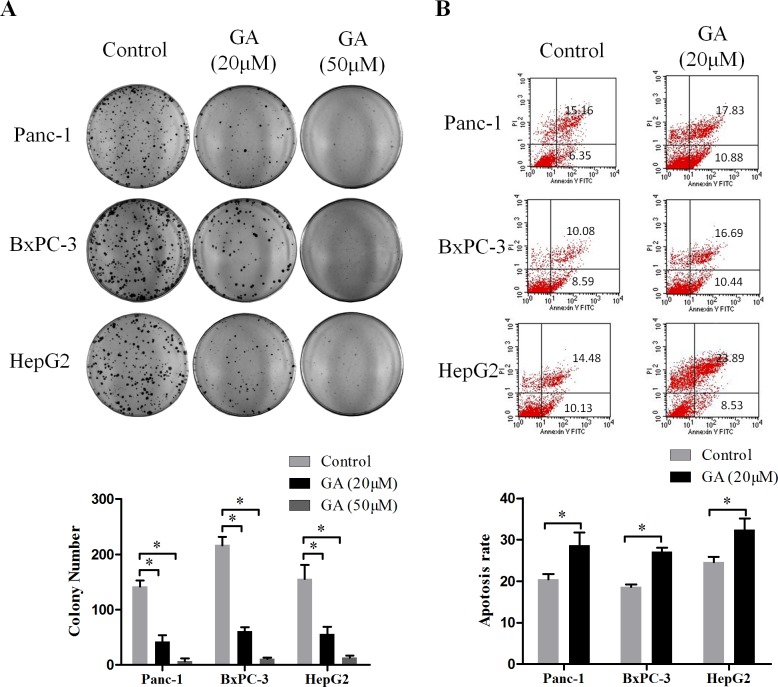
GA treatment inhibits the clone formation and induces apoptosis of cancer cells (**A**) The effects of GA on the colony forming ability of Panc-1, BxPC-3, and HepG2 cells. Images are representative of three independent experiments. (**B**) The effects of GA on cancer cells apoptosis was detected by flow cytometry. *P < 0.05.

Next, we detected the effect of GA on clone formation capability of cancer cells Panc-1, BxPC-3, and HepG2. As shown in Figure 2A, treatment with 20μM GA markedly decreased the number of colonies compared to the untreated control cells; moreover, there were almost no clone forming under the GA concentration of 50μM.

To estimate the percentage of apoptotic cell death induced by GA, the flow cytometric analyses were conducted after Panc-1, BxPC-3, and HepG2 cells treated with or without GA (20μM) for 48h. As shown in Figure 2B, treatment of cancer cells with GA caused an increase in apoptotic population as compared to the untreated control cells.

These results demonstrated that GA has a potent effect to against clone formation and induce apoptosis of cancer cells.

### GA inhibits the migration and invasion of cancer cells

**Figure 3:**
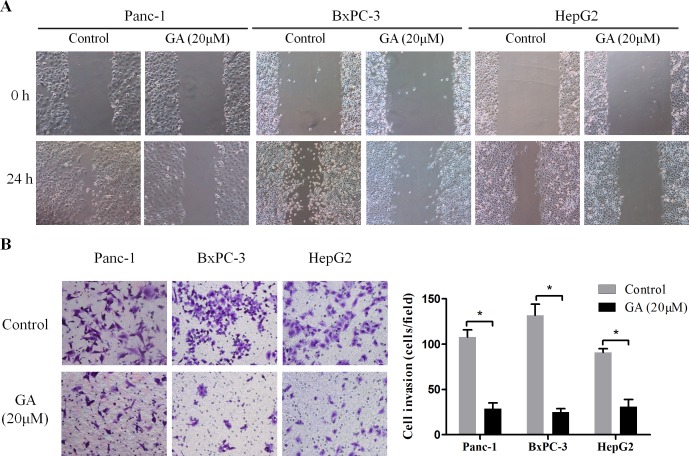
GA treatment inhibits the migration and invasion ability of cancer cells (**A**) Wound-scratch assay were performed in Panc-1, BxPC-3, and HepG2 cells pretreated with 20μM GA or not. Images were visualized at 0 h and 24h at a magnification of 100×. (**B**) The effects of GA on the invasion ability of cancer cells were assessed by Matrigel-invasion assay. Images are representative of three independent experiments. *P < 0.05.

Invasion and metastasis are the most important hallmarks of malignant cancer cells. To explore the effect of GA on the migration ability of cancer cell lines Panc-1, BxPC-3, and HepG2, wound-scratch assay was performed under serum-free conditions. As shown in Figure 3A, the migration ability of cancer cells was impaired by GA (20μM) intervention compared to the untreated control cells. Additionally, a Matrigel invasion assay was conducted to investigate the effect of GA on the invasion ability of cancer cells. Consistently, a significantly decreased invasion was observed in Panc-1, BxPC-3, and HepG2 cells treated with GA at a concentration of 20μM as compared with untreated control cells in each group (Figure 3B). These findings suggest that GA inhibits the migration and invasion capacities of cancer cells *in vitro*.

### GA prevents lipogenesis of cancer cells

**Figure 4:**
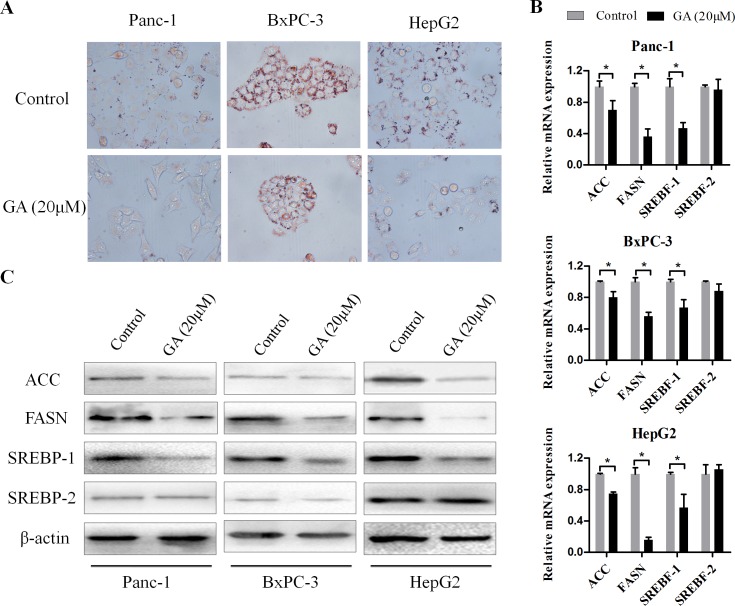
GA prevents lipogenesis of cancer cells (**A**) Oil Red O staining was used to visualized the lipid droplets changes in Panc-1, BxPC-3, and HepG2 cells pretreated with 20μM GA or not. (**B**) The effects of GA on the mRNA expression of lipogenic genes (ACC, FASN, SREBF-1 and SREBF-2) were examined by real-time PCR with β-actin as the normalized reference gene. *P < 0.05. (**C**) The effects of GA on the protein expression of lipogenic genes were examined by Western blotting analysis using β-actin as an internal loading control.

Increased lipogenesis has been proposed to play a key role in cancer cell survival and progression [15]. To determine whether GA has an influence on lipid metabolism of cancer cells, Panc-1, BxPC-3, and HepG2 cells were treated with GA (20μM) for 48h, then Oil red O staining was performed. As shown in Figure 4A, the content of lipid drops in cancer cells was substantially reduced after GA intervention. The regulation on fatty acid synthesis or lipogenesis involves modulation of multiple lipogenic genes at both transcriptional and post-transcriptional level. Therefore, we wanted to determine whether GA-induced reduction of lipid drops in cancer cells is mediated by regulating expression of key lipogenic genes, e.g., acetyl-CoA carboxylase (ACC), fatty acid synthase (FASN), sterol regulatory element binding transcription protein (SREBP)-1, and SREBP-2. To test this, we measured the expression of lipogenic genes by qRT-PCR (Figure 4B). GA treatment of Panc-1, BxPC-3, and HepG2 resulted in a dramatically decrease in ACC, FASN, SREBP-1 (SREBF-1) expression at mRNA levels. Among them, the reduction change on FASN expression is the most obvious. However, we did not see notable change on SREBP-2 (SREBF-2) expression at transcriptional level after GA treatment for 24h. And these observations were confirmed at the protein level by immunoblotting (Figure 4C). Using immunofluorescence, we further confirmed that the expression of FASN in three cancer cell lines was markedly suppressed by GA intervention (Supplementary Figure 1). Together, these data indicated that GA prevents lipogenesis of cancer cells via inhibiting lipogenic genes ACC, FASN, and SREBP-1.

### Knockdown of AMPK rescues GA-induced suppression of lipogenesis of cancer cells

**Figure 5:**
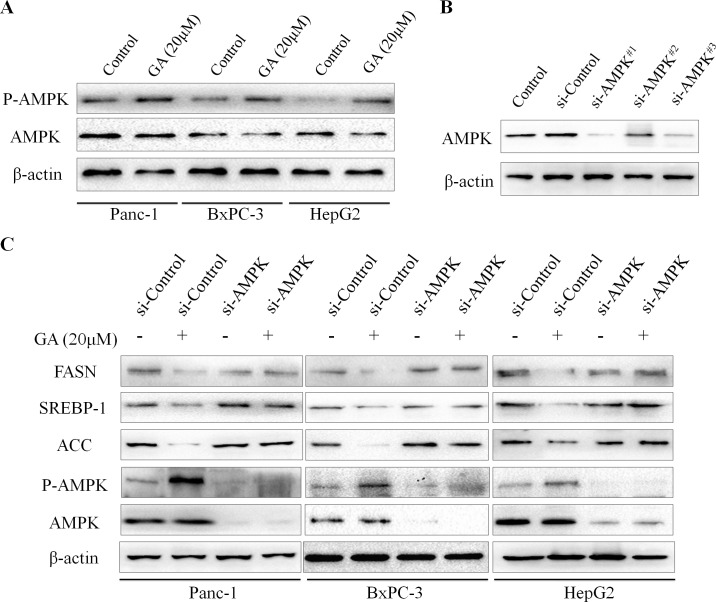
GA inhibits the expression of lipogenic genes via activating AMPK signaling (**A**) The effects of GA on the activity of AMPK in Panc-1, BxPC-3, and HepG2 cells were measured by Western blotting analysis. (**B**) The efficiency of siRNAs targeting AMPK in BxPC-3 cells was evaluated by Western blotting. (**C**) Immunoblotting results revealed that knocking down AMPK expression restored GA-prevented lipogenic genes expression in cancer cells. β-actin was used as an internal loading control.

Previous studies have established the AMP-activated protein kinase (AMPK) as an upstream regulator of genes involved in lipid metabolism [16]. The activation of AMPK leads to the suppression of lipogenesis. Based on the above promising findings, we speculated that the effect of GA on cancer cell lipogenesis may mediated by AMPK signaling. To test this hypothesis, we further examined the effect of GA on the activity of AMPK signaling. Immunoblotting results revealed that the phosphorylation level of AMPK (P-AMPK) in cancer cells was significantly increased in response to GA treatment (Figure 5A). To verify GA-inhibited lipogenesis in cancer cells is mediated by AMPK signaling, siRNA technology was developed to knockdown AMPK expression. Three siRNA sequences were designed and the efficiency of these siRNAs was detected by immunoblotting (Figure 5B). We then chose si-AMPK^#1^ for further experiments for its excellent effect to knockdown AMPK expression. We found that knocking down AMPK expression alone didn't affect the expression of FASN, SREBP-1, and ACC in Panc-1, BxPC-3 and HepG2 cells (Figure 5C); however, GA prevented these proteins expression was restored by AMPK knockdown (Figure 5C). Together, these data suggested that AMPK signaling is involved in GA suppressed lipogenesis in cancer cells.

### GA suppresses the tumor growth *in vivo*

**Figure 6:**
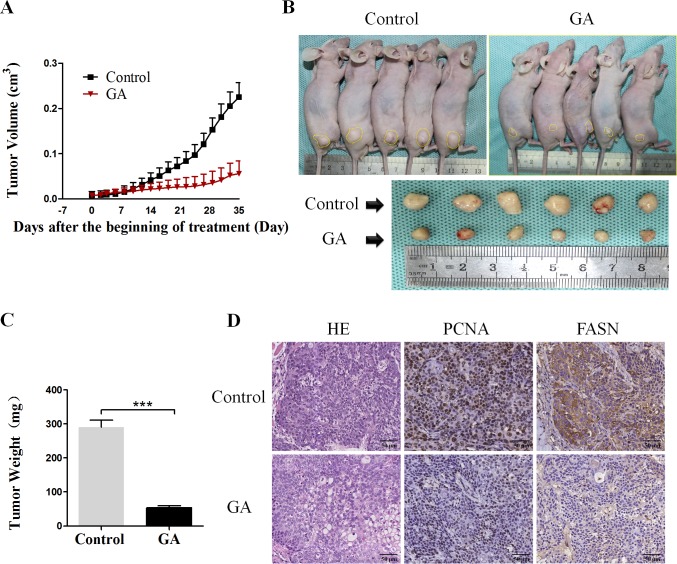
GA prevents the subcutaneous xenograft tumor growth and the expression of lipogenic enzyme *in vivo* (**A**) The tumors volume was calculated every 3 days throughout the experiment. The tumor growth was dramatically retarded by GA administration. (**B**) Representative photograph of subcutaneous xenograft tumors derived from mice in GA group and Control group. (**C**) The average tumor weights in GA group and Control group were measured at the end of the experiment. *P < 0.001. (**D**) Representative image of H&E staining and immunohistochemical staining for PCNA and FASN in GA group and Control group.

Based on the above *in vitro* findings, we next conducted *in vivo* experiment to confirm the effect of GA on cancer cells. 100μL BxPC-3 and PSCs mixed single-cell suspension (cell proportion 5:1, containing 1×10^6^ cancer cells) was injected to the right limb subcutaneous of BALb/c nude mice. After one week, mice were randomly divided into two cohorts, one of which received vehicle and the other administrated with GA as described in the Materials and Methods. The tumor volume was monitored, as shown in Figure 6A, the tumor growth in GA group was dramatically retarded as compared with it in Control group. At the end of the experiment, the average tumor volume and tumor weight of the group treated with GA was significantly lower compared with that of the Control group (Figure 6B and 6C). Consist with *in vitro* studies, the immunohistochemistry results showed that the proliferation of cancer cells was inhibited by GA administration as there was less and weak expression for PCNA in GA group compared with that in Control group (Figure 6D). Moreover, the tumor tissues from mice in GA group exhibited low level of FASN staining compared with that from mice in Control group (Figure 6D). And this was confirmed by immunoblotting (Supplementary Figure 2). FASN expression in the GA-treated group was obviously decreased compared to the Control group. In addition, the phosphorylation level of AMPK (P-AMPK) in the GA-treated group was significantly increased compared to the Control group. These data showed that GA can suppress the *in vivo* tumor growth through activating AMPK signaling and inhibiting critical members involved in lipogenesis.

## DISCUSSION

Cancer metabolism has recently become a hotspot in the field of cancer research [17]. Recent research has demonstrated that cancer cells often bear a metabolism characteristic distancing from that of normal cells [18]. This metabolic rewiring in cancer cells involves aberrantly activated aerobic glycolysis and enhanced *de novo* lipid biosynthesis that was driven by metabolism related enzymes or genes [19]. In this view, to find drugs targeting the key enzymes or genes involved in cancer cell specific metabolic pathways may have the potential to disrupt tumor cell proliferation and survival without affecting normal cells, thus providing a powerful new intervention to treat cancer.

Elevated *de novo* lipogenesis is one of the most important hallmarks of altered metabolism in pancreatic cancer and liver cancer and has been proposed to be a new drug target for the development of anti-cancer agents [4]. A previous study has indicated that increased lipogenesis is associated with development and progression of human hepatocellular carcinoma [20]. The newly synthesized lipid can be used for building cell membrane during cell proliferation and providing fuel for rapid growth cancer cell when demanded. Recently, increasing evidence suggests that the lipid may play important roles in signaling transduction associated with tumor growth [21; 22]. Overexpression of genes encoding lipogenic enzymes was responsible for the elevated lipid biosynthesis in cancer [23]. Among them, ACC and FASN are two important enzymes during cell *de novo* lipogenesis. The significant role of FASN and ACC in cancer development has been well established in the past [24; 25]. Elevated expression of FASN and ACC in cancer cells is related to markedly worse prognosis in many human cancers, including pancreatic cancer [4]. Inhibit the expression of FASN and ACC by metformin prevents liver tumorigenesis [26]. In this study, our results demonstrated that GA possesses a potent inhibitory effect on cancer cell growth both *in vitro* and *in vivo*. Moreover, *de novo* lipid synthesis in cancer cells was dramatically attenuated by GA. Inhibition of critical genes involved in lipid synthesis could be the basis of these effects induced by GA.

SREBPs are a family of transcription factors that regulate lipid homeostasis by regulating the expression of the core and rate-limiting enzymes involved in lipid synthesis, including ACC and FASN [27]. Three SREBP isoforms, SREBP-1a, SREBP-1c and SREBP-2, have been identified in mammalian cells. Among them, SREBP-1a and SREBP-1c were encoded by a same gene SREBF-1 while SREBP-2 was encoded by SREBF-2. The biological function of SREBP-1 is to control the lipogenic gene expression while SREBP-2 to regulate the cholesterogenic gene expression. It has been reported that elevated expression of SREBP-1 has been detected in several cancer types and was closely correlated with malignant transformation, cancer progression and metastasis [28]. Inhibit the expression of SREBP-1 leaded to impaired tumor growth [23]. The studies we described here showed that the expression of SREBP-1 was reduced by GA treatment in pancreatic cancer cells and hepatocellular carcinoma cells, however, the expression of SREBP-2 remained unchanged. These results indicated GA may only have an effect on lipid metabolism and has no effect on cholesterol metabolism.

The cellular energy sensor AMPK is a highly conserved Ser/Thr protein kinase complex that plays a crucial role in the regulation of cellular energy metabolism [29]. Previous study has established AMPK as an upstream regulator of both lipid and glucose metabolism [30]. The phosphorylation of AMPK resulted in reduced lipid biosynthesis via inhibiting the expression of SREBP-1c and its target genes, ACC and FASN [31]. Consistent with this, our study showed that GA significantly downregulated the expression of genes related to lipid metabolism accompanied with AMPK activation. And knockdown AMPK rescued these effects induced by GA.

GA has received much attention for its multitude biological activities. The purification of GA from *Ginkgo biloba* Leaves has been effectively improved. Previous study indicated that GA may serve as a promising agent to inhibit HIV protease activity and HIV infection *in vitro* [11]. In addition, the molluscicidal property of GA has been examined [13]. Recently, the antitumor efficacy of GA has cause increasing attentions. Aberrant SUMOylation has been found to be implicated in the development of cancer. It was reported that GA inhibit protein SUMOylation both *in vitro* and *in vivo* [32]. Furthermore, Zhou et al. showed that GA reduced the viability of various types of cancer cells in a manner of inhibiting division, retarding the progress of cell cycle and inducing apoptosis without affecting the viability of non-tumorigenic cells [14]. Consistently, the present study demonstrated that GA suppressed the proliferation, migration, and invasion as well as *de novo* lipogenesis of cancer cells. Inducing the activation of AMPK and inhibiting the critical genes involved in lipogenesis may be the underlying mechanisms. More importantly, GA has a relatively safe toxicity profile even at high concentrations which is fatal to tumor cells.

## CONCLUSION

In conclusion, our data provide evidence that GA can serve as a safe and potent anti-tumor agent to against pancreatic cancer through regulating signaling pathway and genes driving lipogenesis in cancer cells. However, whether other mechanisms is involved in the anti-tumor effects of GA and whether GA has a synergistic effect with other chemotherapy drugs warrant further study.

## MATERIALS AND METHODS

All experimental protocols were approved by the relevant Ethical Committee of the First Affiliated Hospital of Medical College, Xi'an Jiaotong University, China.

### Cell culture and reagents

The human pancreatic cancer cell lines Panc-1 and BxPC-3 were purchased from the Cell Bank of the Chinese Academy of Sciences (Shanghai, China) and maintained as previously described. HepG2 (human hepatocellular carcinoma cell line), HL-7702 (human normal hepatocyte) and HUVEC were kindly provided by Dr. Chang Liu (Medical College, Xi'an Jiaotong University) and cultured as per their instructions. GA (C15:1; C_22_H_34_O_3_; molecular weight: 346.50), Oil Red O, and MTT (3-(4,5-dimethyl-2-thiazolyl)-2,5-diphenyl-2-H-tetrazolium bromide) were purchased from Sigma (St. Louis, MO, USA). GA was initially dissolved in pure methanol at the stock concentration of 1mM. Working dilutions for GA were made in culture medium immediately before use with 10μM of methanol used as control in all experiments. The antibodies used in this study are listed in Supplementary Table 1.

### Cell viability assay

Cancer cell lines(Panc-1, BxPC-3 and HepG2) and two normal cell lines(HL-7702 and HUVEC) were plated into 96-well plates at a density of 5 ×10^3^ cells per well and treated with various concentrations (0, 1, 2, 5, 10, 20, 50, and 100μM) of GA. At the indicated time points (12, 24, 36, and 48h), cell viability was assessed by the MTT assay and the absorbance was measured at 490 nm using a multi-well microplate reader (BIO-TEC Inc, VA).

### Apoptosis assay

Cell apoptosis was assessed by flow cytometry with an Annexin V-FITC/PI apoptosis detection kit (Beyotime Instituteof Biotechnology, Shanghai, China) according manufacturer's instructions. Briefly, Cancer cells were seeded into 6-well plates a density of 1×10^5^ cells per well, after starved overnight, cells were treated with fresh medium containing various concentrations (0μM and 20μM) of GA for 48h. Then cells were trypsinized, washed with PBS, and stained with Annexin V and propidium iodide (PI). The percentage of apoptotic cells was quantified by flow cytometry using a FACSCalibur (BD Biosciences, USA) instrument. The total apoptosis rate was calculated by summing the rate of populations stained with annexin V-FITC^+^/PI^−^ (early apoptotic cells) and Annexin V-FITC^+^/PI^+^ (late apoptotic cells).

### Wound-scratch assay

Wound-scratch assay was performed to detect the migration ability of cancer cells. Briefly, Cancer cells were serum-starved overnight and then pre-treated with GA in 6-well plates for 24h. The monolayers were then scratched with a 200μl sterile pipette tip. Floating cells were washed off with PBS, and the adherent cells were maintained in serum-free media. Images of the same fields were acquired by microscope (Nikon Instruments Inc.) at a magnification of 100× at two preselected time points (0 h and 24 h).

### Matrigel-invasion assay

For cell invasion assessment, Transwell chamber assays were performed using Transwell chambers (Millipore, USA) according to a protocol described previously [33]. In brief, cancer cells were serum-starved overnight and then pre-treated with GA for 24h. Then cells were suspended and added to the upper chamber of the Transwell chambers that were coated with Matrigel (BD Biosciences, USA) before experiment. The non-invasive cells were slightly removed from the upper surface by a cotton-tipped swab after 24h incubation. Invading cells on the bottom surface of the filter were fixed and then stained with 0.1% crystal violet. The invading cell numbers were quantified by counting the stained cells under microscope (Nikon Instruments Inc.) at a magnification of 200×.

### RNA interference

To knockdown AMPK expression, three AMPK-specific siRNAs and one negative control siRNA were designed and synthesized by GenePharma Co., Ltd (Shanghai, China). The siRNA sequences are provided in Supplementary Table 2. The transfection was performed as previously described [33]. The cells were used for subsequent experiments 24 h after transfection.

### Colony formation assay

1000 Cells were seeded into 35-mm petri dish and allowed overnight to adhere. The next day, GA (final concentration is 20μM) was added to the dishes for 24 h following which media was replaced with drug free media. Cells were further cultured for 2 weeks to allow colonies to form. At indicated time point, colonies were fixed with 4% paraformaldehyde and then stained with 0.1% crystal violet solution, rinsed and then imaged. And the number of colonies > 0.5mm in diameter was counted by using a microscope (Nikon Eclipse Ti-S, Japan) at magnification of 40×.

### Oil Red O staining

The Oil Red O staining was used to visualize the lipid droplets in cancer cells. Cells were washed with PBS and fixed in 4% paraformaldehyde for 1 h, and then stained with pre-warmed 0.25% Oil Red O working solution (0.5% Oil Red O was diluted with propylene glycol for long-term storage, and working dilutions were made immediately before use with ddH_2_O) for 15 minutes in 60 ºC oven. After being washed twice with PBS, the cells were photographed under the light microscope (Nikon Eclipse Ti-S, Japan) at magnification of 200×.

### Quantitative real-time PCR

Total cell RNA was extracted using Trizol reagent (Invitrogen, CA, USA) according to the manufacturer's instructions. Then cDNA synthesis was performed using a PrimeScript RT reagent Kit (TaKaRa, Dalian, China). Real-time PCR was performed with an iQ5 Multicolor Real-Time PCR Detection System (Bio-Rad, Hercules, CA, USA) using a SYBR Green PCR Kit (TaKaRa) according to the manufacturer's instructions. The amplification consisted of predenaturation at 94°C for 4 min, denaturation at 94°C for 30 s, annealing at 60°C for 30 s and extension at 72°C for 30 s for 40 cycles. The primer sequences used are listed in Supplementary Table 3. The ΔΔCT method was used to calculate the Relative expression of the sample genes with β-actin as the normalized reference gene.

### Western blotting analysis

Total proteins were extracted by RIPA Lysis Buffer (Beyotime, Guangzhou, China) and the concentration of proteins was determined using the BCA protein assay kit (Pierce, Rockford, USA) according to the Manufacturer's instruction. Then Western blotting assay was performed as previously described [34]. The protein expression was visualized with the enhanced chemiluminescence (Millipore, USA). Images were captured using the ChemiDoc XRS imaging system (Bio-Rad, USA) and Quantity One image software was used for the densitometry analysis of each band. β-actin was used as an internal loading control.

### Animal experiments and histological analyses

Twenty 6-week Male BALb/c nude mice were supplied by and housed in the Animal Center at Medical College, Xi'an Jiaotong University. Animal experiments were conducted according to the ethical guidelines established by the relevant Ethical Committee of the First Affiliated Hospital of Medical College, Xi'an Jiaotong University. 5 × 10^5^ BxPc-3 cells mixed with 1 × 10^5^ PSCs (PSCs were isolate and cultured as previously described) were resuspended in 30μl HBSS and then injected to the subcutaneous of the right back of nude mice. After one week, animals were randomly divided into two groups, the first group received vehicle (100μl saline) by oral gavage (Control group, n = 10); the other group was administered with GA (suspended in saline, 50 mg/kg) via gastric gavage daily for 4 weeks (GA group, n=10). The tumors’ dimensions were monitored with vernier calipers every 2 days throughout the experiment, and the tumor volume was calculated using the following formula: V (tumor volume) = d (shorter diameter) 2 ×D (longer diameter) ×0.5. At the end of the experimental period of 6 weeks, the mice were euthanized and individual tumor weights were measured. The tumor samples were fixed with 4% paraformaldehyde and embedded in paraffin. Serial sections of 4 mm were cut for hematoxylin and eosin (H&E) staining and immunohistochemical staining as previously described [35].

### Statistical analysis

The results are expressed as means ± standard deviation (SD). One-way analysis of variance (ANOVA) was used to evaluate the statistical significance between groups with Dunnett's test for post-hoc analysis, using SPSS (version 15.0; SPSS, Chicago, IL, USA). P value < 0.05 was considered to be statistically significant. Each experiment was performed at least for three times.

## SUPPLEMENTARY MATERIAL FIGURES AND TABLES


