# Supplementary material for: Ginkgolic acid suppresses the development of pancreatic cancer by inhibiting pathways driving lipogenesis
Source: Oncotarget. 2015 Mar 26;6(25):20993–1003. doi: 10.18632/oncotarget.3663 (PMC4673245; doi:10.18632/oncotarget.3663)
Supplement: Supplementary file 1 [file oncotarget-06-20993-s001.pdf]

Ginkgolic acid suppresses the development of pancreatic cancer by inhibiting pathways driving lipogenesis

Supplementary Material

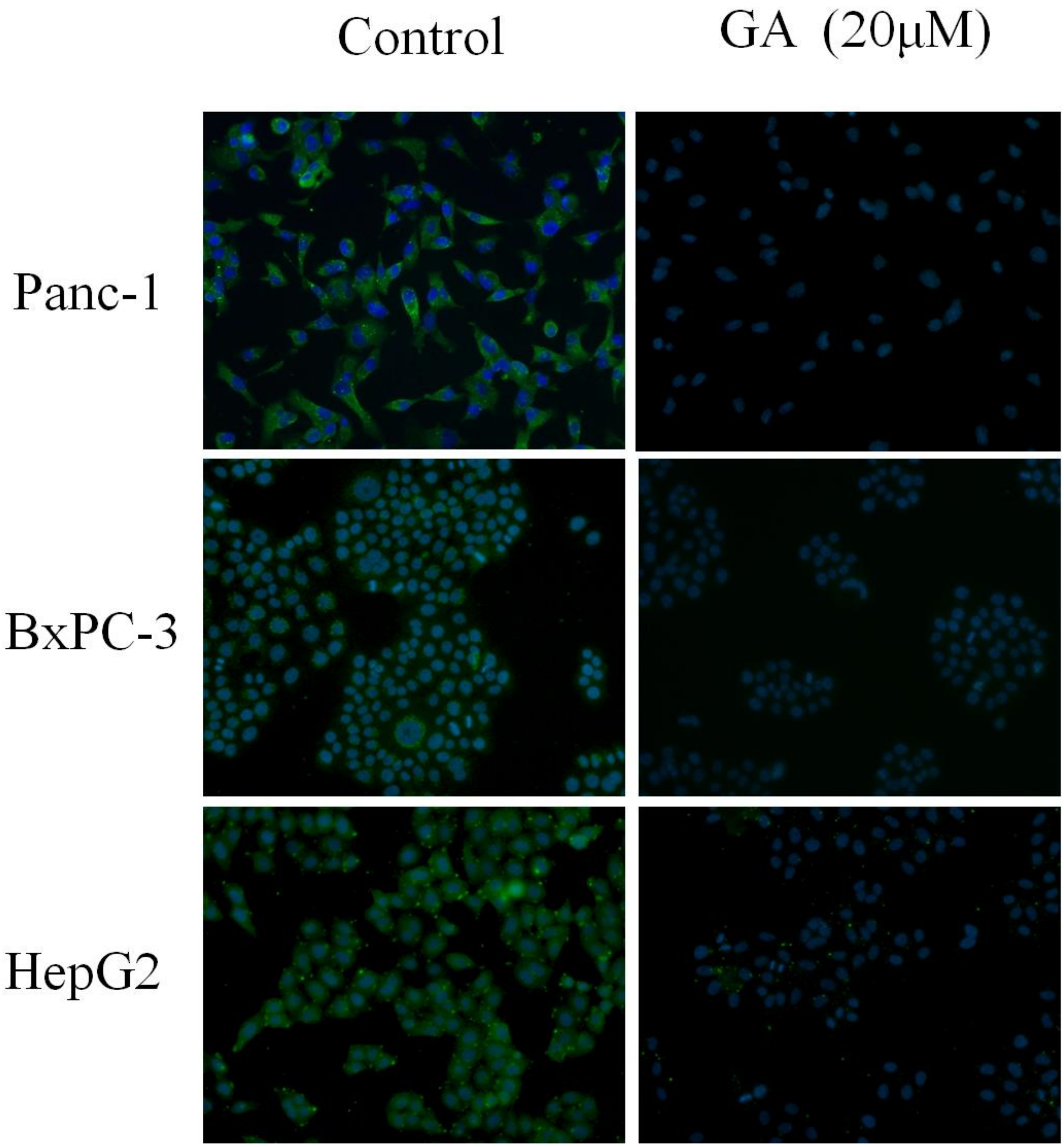

Supplementary Figure 1: Representative images of immunofluorescence for FASN protein in Panc-1, BxPC-3, and HepG2 cells treated with GA or not. Magnification, × 200.

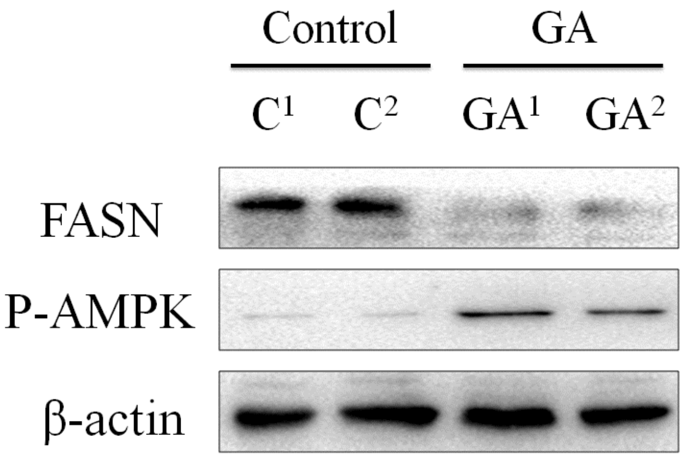

Supplementary Figure 2: Western blotting analysis of FASN and P-AMPK expression in subcutaneous xenograft tumor specimens from Control group and GA-treated group. The expression of FASN was inhibited by GA administration accompanying with increased AMPK phosphorylation. C^1 and C^2 represent two different tumor samples from Control group, respectively; GA^1 and GA^2 represent two different tumor specimens from Control group.

Supplementary Table 1: A list for the utilized primary antibodies

Antibody	Catalog#	Dilution & Use	Company
Rabit anti-FASN	ab128870	1:1000(WB)<br>1:150(IHC/IF)	Abcam
Mouse anti-SREBP-1	Ab3259	1:1000(WB)	Abcam
Mouse anti-SREBP-2	Ab28482	1:1000(WB)	Abcam
Rabbit anti-PCNA	10205-2-AP	1:100(IHC)	Proteintech
Rabit anti-ACC	#3662	1:800(WB)	Cell Signaling Technology
Rabbit anti-AMPK	#2532	1:1000(WB)	Cell Signaling Technology
Rabbit anti-P-AMPK	#2535	1:750(WB)	Cell Signaling Technology
Mouse anti-β-actin	sc-47778	1:1500(WB)	Santa Cruz Biotechnology
Goat anti-rabbit IgG-HRP	sc-2004	1:10000(WB)	Santa Cruz Biotechnology
Goat anti-mouse IgG-HRP	sc-2005	1:10000(WB)	Santa Cruz Biotechnology

Supplementary Table 2: siRNA Sequences for AMPK

Human genes	siRNA sequences
si-AMPK#1	Sense: 5'-UUCUCCGAACGUGUCACGUTT-3'<br>Antisense: 5'-ACGUGACACGUUCGGAGAATT-3'
si-AMPK#2	Sense: 5'-CGGGAUCAGUUAGCAACUATT-3'<br>Antisense: 5'-UAGUUGCUAACUGAUCCCGTT-3'
si-AMPK#2	Sense: 5'- GCGUGUACGAAGGAAGAAUTT-3'<br>Antisense: 5'- AUUCUUCCUUCGUACACGCTT-3'
si-Control	Sense: 5'- GAGGAGAGCUAUUUGAUUATT-3'<br>Antisense: 5'-UAAUCAAAUAGCUCUCCUCTT -3'

Supplementary Table 3: Primers for real-time PCR

Genes	Primer Sequences	Products Size (bp)
FASN	**Forward:** 5'- AACGGCAACCTGGTAGTGAG-3'<br>**Reverse:** 5'- GTGTCCATGAAGCTCACCCA-3'	251
ACC	**Forward:** 5'- GCTGCTCGGATCACTAGTGAA-3'<br>**Reverse:** 5'- TTCTGCTATCAGTCTGTCCAG-3'	339
SREBF-1	**Forward:** 5'- GATGCGGAGAAGCTGCCTAT-3'<br>**Reverse:** 5'- GCTGTGTTGCAGAAAGCGAA-3'	234
SREBF-2	**Forward:** 5'- CGGGCGCAACGCAAACAT-3'<br>**Reverse:** 5'- GTGACCTGGGTGAATGACCG-3'	437
β-actin	**Forward:** 5'-CATCACTATCGGCAATGAGC-3'<br>**Reverse:** 5'- GACAGCACTGTGTTGGCATA-3'	159
